# Test−Retest Reliability of Isokinetic Ankle, Knee and Hip Strength in Physically Active Adults Using Biodex System 4 Pro

**DOI:** 10.3390/mps6020026

**Published:** 2023-03-09

**Authors:** Juho Tuominen, Mari Leppänen, Heidi Jarske, Kati Pasanen, Tommi Vasankari, Jari Parkkari

**Affiliations:** 1 Facualty of Medicine and Health Technology, Tampere University, 33014 Tampere, Finland; 2 Tampere Research Center of Sports Medicine, UKK Institute for Health Promotion Research, 33500 Tampere, Finland; 3 Tampere University Hospital, 33520 Tampere, Finland; 4 Sport Injury Prevention Research Centre, Faculty of Kinesiology, University of Calgary, Calgary, AB T2N 1N4, Canada; 5 Alberta Children’s Hospital Research Institute, University of Calgary, Calgary, AB T2N 1N4, Canada; 6 McCaig Institute for Bone and Joint Health, University of Calgary, Calgary, AB T2N 1N4, Canada; 7 Faculty of Sport and Health Sciences, University of Jyväskylä, 40014 Jyvaskyla, Finland

**Keywords:** isokinetic, dynamometry, Biodex, test−retest, reliability, ankle, knee, hip

## Abstract

Background: The isokinetic dynamometry is considered a gold standard in muscle strength testing. The reliability of lower limb isokinetic strength measurements has not been thoroughly evaluated. Objective: To examine the test−retest reliability of isokinetic ankle plantar and dorsiflexion, ankle inversion and eversion, knee extension and flexion and hip abduction and adduction strength in physically active adults using Biodex System 4 Pro. Methods: Peak torques (PTs) and average peak torques (APTs) of the dominant and nondominant lower limbs were tested twice in 19 physically active adults 7 to 14 days apart. Results: The intraclass correlation coefficients (ICC) values varied from excellent to moderate and coefficient of variation of typical error (CV_TE_) values were 6.6–19.5%. Change in the mean expressed as a percent varied from −3.1% to 9.6%. There was no difference in the reliability between PT and APT values. Dominant lower limb was more reliable in every case if there was difference between limbs. Conclusion: Test−retest reliability of isokinetic ankle, knee and hip strength in physically active adults using Biodex System 4 is mostly good or excellent. However, the observed range of the random variation has to be noted when using it in scientific follow-up studies or evaluation of patient progress in clinical settings.

## 1. Introduction

The Biodex System 4 Pro (Biodex Medical System Inc, Shirley, NY, USA) is a multi-mode computerized robotic dynamometer which is used in sports and orthopedic medicine, pediatric medicine, neurorehabilitation, geriatrics, industrial medicine and research. The dynamometer makes it possible to measure force production capabilities in different muscle groups [1]. Isokinetic dynamometry is accepted as the gold standard for the estimation of muscle strength [2]. The main purposes of isokinetic testing are to determine muscle performance, to follow progress and to examine imbalance between body sides and agonist–antagonist muscle relations. The reliability of the dynamometers is a key factor in this context.

A high number of studies have assessed the between-session reliability of the knee extension and flexion strength measurements, both using older versions of the Biodex as well as Biodex System 4 Pro. These studies have reported moderate to excellent, mainly excellent reliability, in peak torques (PTs) [3,4,5,6,7,8,9,10,11,12,13,14,15,16,17,18]. The reliability of the average peak torques (APTs) in knee measurements has been less studied, but results have been so far excellent [3,16]. Although the reliability of the knee measurements has been well established, a smaller number of studies have evaluated the reliability of ankle and hip strength measurements [3,4,5,6,7,19] and very few of them use the Biodex System 4 Pro. Hence, the reliability of lower limb isokinetic strength measurements has not yet been thoroughly evaluated.

To our knowledge, the reliability of the ankle strength measurements has not been previously studied using the Biodex System 4 Pro. Previous studies using the earlier versions of the Biodex have reported excellent reliability for the ankle dorsiflexion strength in PT and in APT [3,5,19]. For the ankle plantar flexion strength, good reliability has been reported in PT and excellent in APT [3,5]. In addition, the reliability of ankle inversion and eversion strength measurements has been reported as being moderate to excellent in PT and APT [4].

Few studies have investigated the reliability of hip abduction and adduction strength measured while lying on one’s side. Maupas and colleagues reported excellent reliability using Biodex System 4 Pro, whereas Meyer and others reported good reliability for abduction and moderate for adduction with older version of Biodex; however, both of these studies only reported on PTs [6,7].

Interestingly, previous reliability studies have focused only on the isokinetic strength of the dominant side; to our knowledge, there is no previous study where both lower limbs, i.e., dominant and nondominant, were examined. Muscle strength is an independent risk factor, e.g., for acute knee injuries; in many risk factor studies, limbs are analyzed separately or compared to each other [20], hence reliability of measuring the strength of the nondominant side needs to be investigated.

For these reasons, the purpose of our study was to examine the test−retest reliability of isokinetic ankle, knee and hip strength of both limbs in physically active adults using Biodex System 4 Pro. Our study adds to knowledge on the reliability of lower limb strength measurements, especially when investigating the ankle and hip in both dominant and nondominant sides, and is of high importance to those using the Biodex System 4 Pro in clinical use or research purposes.

## 2. Materials and Methods

### 2.1. Participants

Nineteen physically active adults (10 men and 9 women; dominant lower limb 16 right and 3 left; age 35.5 ± 10.5 years; body mass index 24.6 ± 3.4 kg/m^2^ [mean and standard deviation]) participated in this study. Exercise backgrounds of the participants were variable but the most common activity was running. The participants did not have any previous experience with isokinetic strength testing. All were non-smokers and non-snuffers, had not reportedly suffered ankle, knee or hip injuries in the last three months and had rest from physical exertion from two days prior to the test and retest. Volunteer participants were recruited via social media application or were asked personally. All participants signed a written informed consent prior to the study. This study has been conducted in accordance with the Declaration of Helsinki.

### 2.2. Procedures

Our testing procedures on measuring ankle, hip and knee strength were based on a pilot study among novice recreational runners [21]. The reliability testing was performed in two sessions (test and retest) 7 to 14 days apart in autumn 2020. Two study assistants conducted all testing sessions on participants. Dominant lower limb was determined by asking participants to kick a ball and step up on a stair. When both tasks were done by the same limb, the limb was determined as a dominant lower limb. If lower limb dominance was not determined by these two tests, the participant was pushed forward lightly by the study assistant. The limb that moved first to maintain balance was determined as the dominant lower limb.

Testing order was the same for all participants and for both testing sessions as follows: (1) ankle plantar/dorsiflexion, (2) ankle inversion/eversion, (3) knee extension/flexion and (4) hip abduction/adduction. Testing was done unilaterally and in a continuous movement for both movement directions. The movements were done by using isokinetic concentric setups. In every movement, the used range of motion was determined by asking the participants to perform their full range of motion whilst keeping the movement comfortable. Each movement was done with both lower limbs. The starting limb was randomized in both testing sessions.

Before both testing sessions, participants were informed about the test protocol and performed a standardized warm-up, including five minutes of walking followed by five minutes of running with a self-selected pace. Prior to every maximal set, participants were allowed to practice the movement with light effort. After they were comfortable with the movement, they did a warm-up including three sub-maximal repetitions with increasing load based on the subjective assessment of the participants (50%, 70% and 90% of their maximal performance). After one-minute rest, participants performed three maximal repetitions. Three repetitions were chosen based on previous studies conducted among novice recreational and youth athletes, which suggest that the subjects without previous experience of isokinetic strength are able to achieve the best peak torque during the three repetitions [20,21,22]. During the maximal sets, participants were verbally encouraged by the test personnel. The practice and warm-up of the contralateral limb or movements started immediately when the dynamometer set-up was changed.

The testing velocity was 30°/s in ankle and hip and 60°/s in knee. Previous studies have shown good to excellent reliability of ankle peak torques using this system at 30°/s [4,5,19]. The lower velocity of 30°/s was also chosen for hip measurements because it was regarded to be more suitable for our novice cohort when measuring torques with small range of motion [21]. The faster angular velocity 60°/s has been used in previous studies to measure knee flexion/extension strength [3,8,10,11,12,13,14,15,20]. The lower limb was weighted and gravitation correction was done in all movement except the inversion/eversion because shaft position was too vertical for accurate gravity correction. Biodex System 4 Pro and System Advantage 4 Software, version 4.63 was used in every test. Force signal was filtered and windowed with the default specifications of the Biodex software.

### 2.3. Test Positions

**Figure 1:**
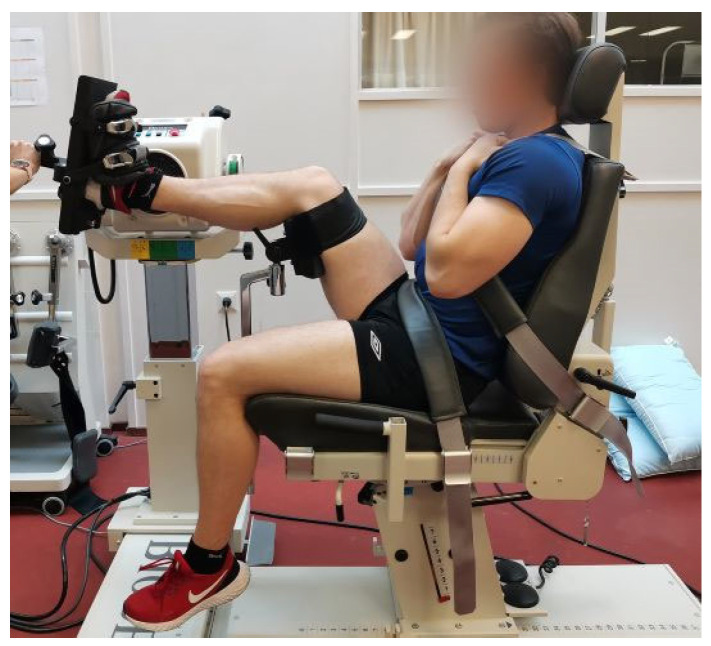
Testing position of ankle plantar and dorsiflexion.

**Figure 2:**
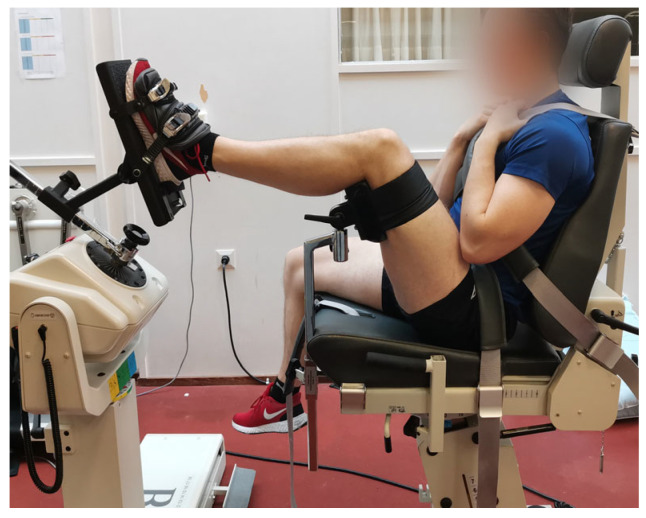
Testing position of ankle inversion/eversion strength measurement.

Test positions were based on the Biodex Multi-Joint System Pro setup/operation manual guidelines and were standardized. The modifications from the manual guidelines were based on practice-based experiences of a system expert and instructor. In both ankle movements, the participants were seated on the chair so that the back of the seat was slightly tilted. Participant’s measured limb was risen and supported on the back of the thigh just above the knee (Figure 1). The shin of the measured limb was set horizontal and straight forward. In the ankle plantar/dorsiflexion measurements, the fibular malleolus was aligned with the axis of the rotation of the dynamometer. The foot was attached to the foot plate. In the ankle inversion/eversion, the foot was attached to the foot plate which was plantar flexed at 20 degrees (Figure 2). The axis of the rotation of the dynamometer was set to pass the body of the talus.

**Figure 3:**
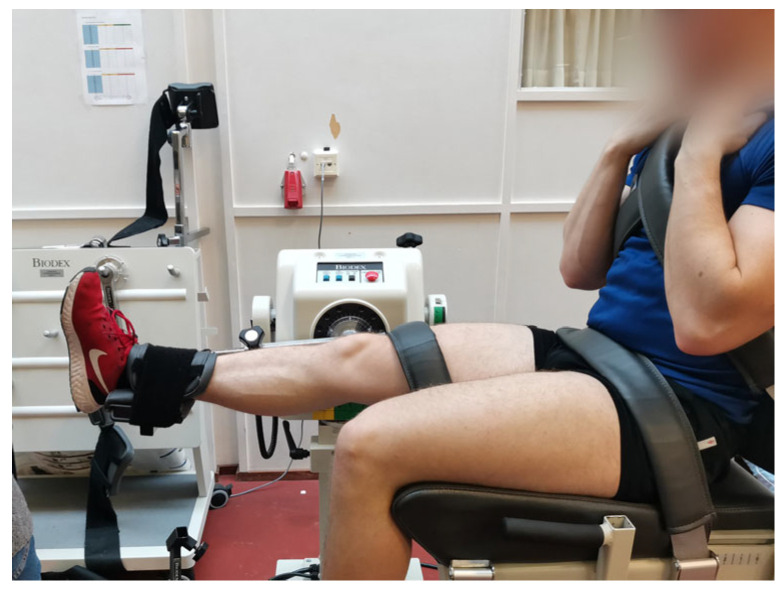
Testing position of knee extension and flexion.

The participants were stabilized by a waist strap and two shoulder straps crossing the participant’s chest in the ankle and knee movement and by a thigh strap in the knee movement. They were asked to hold on to the shoulder straps in both ankle and knee movements. In the knee extension/flexion measurement, the participants were seated on the chair in a comfortable position and the femur was fully supported by the chair seat (Figure 3). The measured limb was straight forward and attached to the dynamometer just above the ankle. The lateral femoral condyle was aligned with the axis of the rotation of the dynamometer.

**Figure 4:**
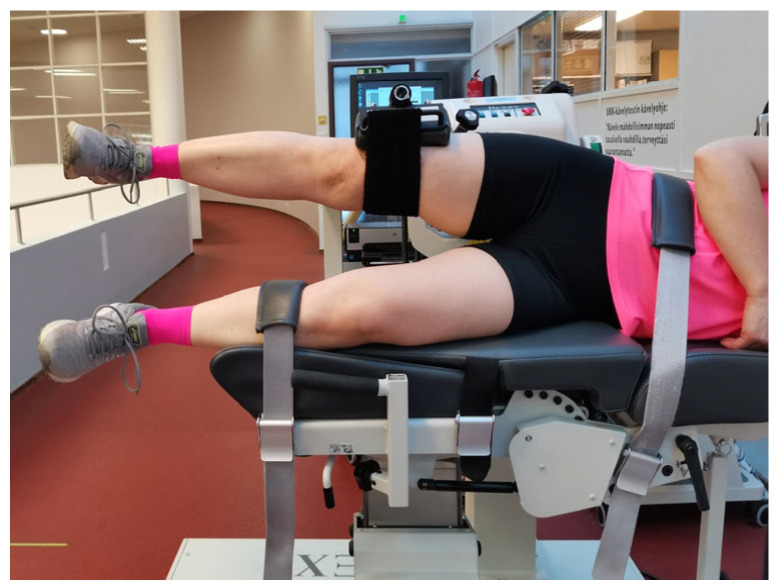
Testing position of hip abduction and adduction.

In the hip abduction/adduction measurement, the participants were lying on their side facing away from the dynamometer and stabilized by a waist strap and a lower limb strap (Figure 4). The greater trochanter of the participants was palpated and utilized to set the axis of the rotation of the dynamometer to align with the axis of the rotation of the measured hip joint. The measured limb was attached to the dynamometer just above the knee (Figure 4). The sampling size varied between movements. If a participant had pain in the limb, for example, those measurements were not performed.

### 2.4. Statistical Analysis

Peak torque (PT) and average peak torque (APT) were chosen as outcome parameters. The PT was defined as highest torque of three repetitions and the mean of the three peak torques was chosen for APT. Mean and standard deviation (SD) of both sessions were calculated. Additionally, the mean difference (DIFF) in normalized absolute values and percentage (DIFF%) were determined. Bland−Altman (BA) plots and 95% limits of agreement (LoA) were visually checked and coefficients of variation of typical error (CV_TE_) were determined [5]. Two-way mixed-effects absolute agreement ICCs with 95% confidence intervals (CI) were used for relative reliability [23]. Reliability values greater than 0.90 were interpreted as excellent, between 0.75 and 0.90 as good, between 0.5 and 0.75 as moderate and less than 0.5 as poor [23]. Statistical analysis was conducted with IBM SPSS Statistics 27 (SPSS Inc, Chicago, IL, USA).

## 3. Results

The ICC values were excellent or good to excellent in all movements except for dominant limb ankle plantar flexion in APT (moderate to excellent), nondominant limb ankle inversion (moderate to excellent) and nondominant limb ankle dorsiflexion (poor to good) (Table 1, Table 2, Table 3 and Table 4).

The CVTE varied between 6.6% and 19.5%, being lowest in dominant limb knee flexion and highest in nondominant ankle dorsiflexion (Table 1, Table 2, Table 3 and Table 4). The LoAs were visually relatively wide.

The DIFF% between test sessions varied from −3.1% to 5.4% except in the PT in dominant limb hip adduction 7.7% and in the PT (8.6%) and APT (9.6%) in dominant limb ankle dorsiflexion (Table 1, Table 2, Table 3 and Table 4).

The difference mean of the BA plots was visually nearby zero except in three movements: in dominant limb APT knee extension, knee flexion and hip adduction.

The reliability between dominant and nondominant limb had some variation. Five test movements did not show difference due lower limb dominance, some movements showed a slight difference and ankle dorsiflexion showed clear difference between dominant and nondominant limb. Dominant lower limb was more reliable in every case if there was difference between lower limbs.

There was no difference in reliability between PT and APT when visually checking results in the tables. BA plots visually did not show heteroscedasticity.

## 4. Discussion

We examined the test−retest reliability of the isokinetic concentric ankle plantar and dorsiflexion, ankle inversion and eversion, knee extension and flexion and hip abduction and adduction strength in physically active adults using Biodex System 4 Pro. The ICCs were mainly excellent in both the dominant and nondominant limb.

The ICC is widely used in the test−retest studies to assess reliability [23,24,25,26]. In the present study, we found good or excellent relative reliability in 30 out of 32 variables. Results reinforced the previous findings that isokinetic strength testing is reliable when analyzing ICCs in movements which have been less studied [3,4,5,6,7,8,9,10,11,12,13,14,15,16,17,18,19]. However, results from different studies should be compared with caution as there are many ways to calculate ICC and it is also affected by subjects´ heterogeneity [23,25].

In the present study, the ankle dorsiflexion strength in nondominant limb showed only moderate reliability. There could be many reasons why this movement was less reliable than the other movements. Participants might have been focusing mainly on the plantar flexion movement and forgetting to produce power in dorsiflexion direction. Some participants also said that it felt difficult to produce power in dorsiflexion direction, which could explain the difference between dominant and nondominant lower limb. Based on these results, we suggest reminding participants to focus on producing power in both directions of the movement when performing the test in future studies or clinical practice.

In our study, the absolute reliability with CV_TE_s was comparable with previous studies describing knee extension, ankle plantar and dorsiflexion strength measurements [5,16]. The level of acceptable CV_TE_s depends on context; in this case, we think it would be advantageous to get lower CV_TE_ values. We also visually checked LoAs and they showed similar results as CV_TE_s and previous studies [3,5,9,12,17]. Both (CV_TE_ and LoA) describe random variation in a measure. The main source of this is usually biological. Participant factors of error might not be focusing on both directions of the movement, mental/motivational changes and normal physical variance between days. Rater-based error sources were variance setting participants on movement set-ups and keeping participants’ movement path correct. Some level error always exists due the apparatus or device, but it is usually unavoidably lumped in with the biological error. We believe that when paying extra attention to the error sources and becoming more experienced with the protocol, it is possible to reduce the amount of error [26].

It is worth noting that we experienced some instability when testing hip movements. Although participants were properly stabilized from their waist and the other lower limb and the measured limb was carefully attached to the dynamometer, the soft tissues of the limb slightly vibrated after producing power and caused multiple peaks in the torque curve. Typically, there were two main peaks and the first one was higher than the other one. The other peaks were clearly smaller and faded along the movement. This problem might have affected the results of the CV_TE_. In spite of these instabilities, our ICC results were excellent in every hip measurement.

As a whole, there did not seem to be a systematic change when examining change in the mean [25]. It was comparable with previous studies and did not show notable difference between test sessions. However, there was a minor change in the mean between test sessions in the hip adduction APT of dominant lower limb [3,4,5,6,7,8,9,10,11,12,13,14,15,16,17,18,19]. This was probably due to random change in the mean which can be called sampling error. Learning effect and desire to improve are factors causing systematic change in the mean [25].

Difference between PT and APT was not visually found when checking results in Table 1, Table 2, Table 3 and Table 4. Similarly, Symons et al. did not find difference between PT and APT [16]. Based on these studies, it is difficult to prefer one over the other.

The dominant side was more reliable than the nondominant in some movements. Dominant limb is typically more developed motorically; therefore, more difficult exercises could be easier to handle by dominant than nondominant limb which might explain the found differences [27]. No previous studies have examined dominant and nondominant lower limbs separately. Based on our study, utilizing the previous reliability study results of the dominant lower limb to the nondominant lower limb should be done with caution.

Knee extension and flexion was the most reliable movement in the present study, whereas ankle plantar and dorsiflexion were the least reliable. This information is new and evidently the strength of this study. There are no previous studies that have made it possible to compare the reliability of the four different movements examined. Another strength is taking into account the opportunity to compare dominant and nondominant lower limbs.

Our study had some limitations which need to be taken into consideration. We had a relatively small number of subjects and the sample size varied in different tests as some subjects were not able to conduct all tests. Although the number of subjects was not large, it was able to show the reliability of the testing device. We were not able to randomize the order of the ankle, hip and knee strength tests, which is not ideal for a reliability study. However, starting limb was randomized in both testing sessions. Another aspect to be noted when interpreting internal and external validity of the results is that our participants were recreational athletes and had a heterogeneous training background. Our participants did not have previous experience with isokinetic strength testing and furthermore were mostly inexperienced with maximal strength tests. They regarded some of the tests, especially the ankle and hip movements, challenging to conduct with maximal effort, hence issues such as participants’ competence and motivation may have influenced our results. Nevertheless, our ICCs were mainly excellent but might have been different with a more homogeneous sample [25], or when examined with athletes or subjects experienced with strength testing.

In conclusion, most of the lower limb isokinetic strength variables measured by the Biodex System 4 Pro achieved good to excellent test-retest reliability in physically active adults. However, the observed range of random variation has to be noticed when using it in the scientific follow-up studies or evaluation of the patient progress in the clinical practice.

## Figures and Tables

**Table 1:** Ankle plantar and dorsi flexion.

		n	Mean1 ± SD	Mean2 ± SD	DIFF	DIFF%	CV_TE_ (%)	ICC (CI 95%)
Ankle Plantar Flexion Dominant	Peak Torque	18	98.7 ± 30.7	98.8 ± 27.5	0.1	0.1	12.7	0.91 (0.75–0.97)
	Average Peak Torque	18	91.0 ± 30.6	93.3 ± 27.8	2.3	2.5	13.8	0.90 (0.73–0.96)
Ankle Plantar Flexion Nondominant	Peak Torque	18	97.5 ± 38.9	97.0 ± 30.1	−0.5	−0.5	12.3	0.94 (0.84–0.98)
	Average Peak Torque	18	90.6 ± 38.2	91.4 ± 29.8	0.8	0.9	12.8	0.94 (0.84–0.98)
Ankle Dorsi Flexion Dominant	Peak Torque	18	27.8 ± 8.3	30.2 ± 7.1	2.4	8.6	8.4	0.93 (0.70–0.98)
	Average Peak Torque	18	26.0 ± 8.2	28.5 ± 7.1	2.5	9.6	8.7	0.93 (0.70–0.98)
Ankle Dorsi Flexion Nondominant	Peak Torque	18	28.4 ± 7.4	29.5 ± 7.9	1.1	3.9	19.5	0.64 (0.02–0.87)
	Average peak torque	18	26.1 ± 7.3	27.3 ± 7.1	1.2	4.6	17.7	0.73 (0.28–0.90)

n = sample size, SD = standard deviation, DIFF = difference between Mean2 and Mean 1, DIFF% = change from Mean1 to Mean2, CVTE (%) = coefficient of variation of typical error, ICC = intraclass correlation coefficients, CI = confidence intervall.

**Table 2:** Ankle inversion and eversion.

		n	Mean1 ± SD	Mean2 ± SD	DIFF	DIFF%	CV_TE_ (%)	ICC (CI 95%)
Ankle Inversion Dominant	Peak Torque	19	31.9 ± 8.4	32.3 ± 7.5	0.4	1.3	9.9	0.95 (0.87–0.98)
	Average Peak Torque	19	29.9 ± 7.8	30.9 ± 7.8	1.0	3.3	9.3	0.93 (0.82–0.97)
Ankle Inversion Nondominant	Peak Torque	18	31.9 ± 9.2	31.3 ± 6.7	−0.6	−1.9	7.7	0.83 (0.55–0.94)
	Average Peak Torque	18	29.8 ± 8.5	29.3 ± 6.2	−0.5	−1.7	13.4	0.84 (0.57–0.94)
Ankle Eversion Dominant	Peak Torque	19	23.6 ± 6.8	23.4 ± 7.8	−0.2	−0.8	13.8	0.95 (0.87–0.98)
	Average Peak Torque	19	22.2 ± 6.6	22.0 ± 7.6	−0.2	−0.9	10.6	0.94 (0.86–0.98)
Ankle Eversion Nondominant	Peak Torque	18	23.1 ± 6.1	23.9 ± 8.0	0.8	3.5	9.9	0.91 (0.75–0.97)
	Average Peak Torque	17	21.3 ± 5.7	22.0 ± 8.3	0.7	3.3	13.7	0.91 (0.75–0.97)

n = sample size, SD = standard deviation, DIFF = difference between Mean2 and Mean 1, DIFF% = change from Mean1 to Mean2, CVTE (%) = coefficient of variation of typical error, ICC = intraclass correlation coefficients, CI = confidence intervall.

**Table 3:** Knee extension and flexion.

		n	Mean1 ± SD	Mean2 ± SD	DIFF	DIFF%	CV_TE_ (%)	ICC (CI 95%)
Knee Extension Dominant	Peak Torque	18	185.4 ± 63.7	187.2 ± 72.3	1.8	1.0	7.0	0.98 (0.95–0.99)
	Average Peak Torque	18	175.2 ± 61.5	180.1 ± 69.8	4.9	2.8	7.6	0.98 (0.94–0.99)
Knee Extension Nondominant	Peak Torque	18	187.2 ± 58.9	190.3 ± 70.5	3.1	1.7	6.9	0.98 (0.95–0.99)
	Average Peak Torque	18	177.9 ± 57.7	181.6 ± 70.1	3.7	2.1	8.5	0.97 (0.93–0.99)
Knee Flexion Dominant	Peak Torque	18	93.7 ± 28.4	98.8 ± 34.2	5.1	5.4	6.6	0.97 (0.91–0.99)
	Average Peak Torque	18	89.2 ± 27.1	93.1 ± 31.7	3.9	4.4	6.8	0.97 (0.93–0.99)
Knee Flexion Nondominant	Peak Torque	18	94.3 ± 30.2	98.1 ± 33.1	3.8	4.0	10.1	0.95 (0.87–0.98)
	Average Peak Torque	18	89.7 ± 29.4	93.0 ± 31.1	3.3	3.7	10.8	0.94 (0.85–0.98)

n = sample size, SD = standard deviation, DIFF = difference between Mean2 and Mean 1, DIFF% = change from Mean1 to Mean2, CVTE (%) = coefficient of variation of typical error, ICC = intraclass correlation coefficients, CI = confidence intervall.

**Table 4:** Hip abduction and adduction.

		n	Mean1 ± SD	Mean2 ± SD	DIFF	DIFF%	CV_TE_ (%)	ICC (CI 95%)
Hip Abduction Dominant	Peak Torque	18	144.4 ± 46.6	143.7 ± 51.0	−0.7	−0.5	10.9	0.95 (0.86–0.98)
	Average Peak Torque	18	131.2 ± 42.6	129.4 ± 40.5	−1.8	−1.4	10.8	0.94 (0.84–0.98)
Hip Abduction Nondominant	Peak Torque	17	149.8 ± 57.8	145.2 ± 49.4	−4.6	−3.1	10.3	0.96 (0.89–0.99)
	Average Peak Torque	17	137.3 ± 52.6	135.2 ± 48.3	−2.1	−1.5	7.6	0.98 (0.94–0.99)
Hip Adduction Dominant	Peak Torque	18	127.3 ± 54.9	133.1 ± 47.9	5.8	4.6	10.5	0.96 (0.90–0.99)
	Average Peak Torque	18	114.5 ± 52.6	123.3 ± 47.2	8.8	7.7	11.1	0.96 (0.88–0.99)
Hip Adduction Nondominant	Peak Torque	17	131.1 ± 50.1	132.8 ± 47.4	1.7	1.3	12.8	0.94 (0.83–0.98)
	Average Peak Torque	17	121.0 ± 49.8	124.1 ± 45.7	3.1	2.6	14.9	0.93 (0.79–0.97)

n = sample size, SD = standard deviation, DIFF = difference between Mean2 and Mean 1, DIFF% = change from Mean1 to Mean2, CVTE (%) = coefficient of variation of typical error, ICC = intraclass correlation coefficients, CI = confidence intervall.

## Data Availability

The data presented in this study are available on request from the corresponding author. The data are not publicly available due to privacy restrictions.
